# Supplementary material for: Afadin couples RAS GTPases to the polarity rheostat Scribble
Source: Nat Commun. 2022 Aug 5;13:4562. doi: 10.1038/s41467-022-32335-8 (PMC9355967; doi:10.1038/s41467-022-32335-8)
Supplement: Supplementary file 1 — Supplementary Information [file 41467_2022_32335_MOESM1_ESM.docx]

# Supplementary Data Legend

**Supplementary Data 1. BioID proteomics-identified proteins with *in vivo* proximity to the short and long isoforms of AFDN.** Preys detected using either AFDN iso1 (l-AFDN) or iso2 (s-AFDN) are listed and are sorted by post-SAINT spectral count. Only hits passing a statistical threshold of FDR≤0.01 were deemed high quality interactions. Gene Ontology (GO) terms describing localization (CC; cellular compartment) and function (BP; biological process) are listed along with GeneIDs, gene names and gene descriptions.

# Supplementary Table Legend

**Supplementary Table 1. Primers used in this study.** Primers for Gateway, Gibson Assembly or restriction site-based cloning as well as CRISPR guides and genotyping.

# Supplementary Figures and Figure Legends


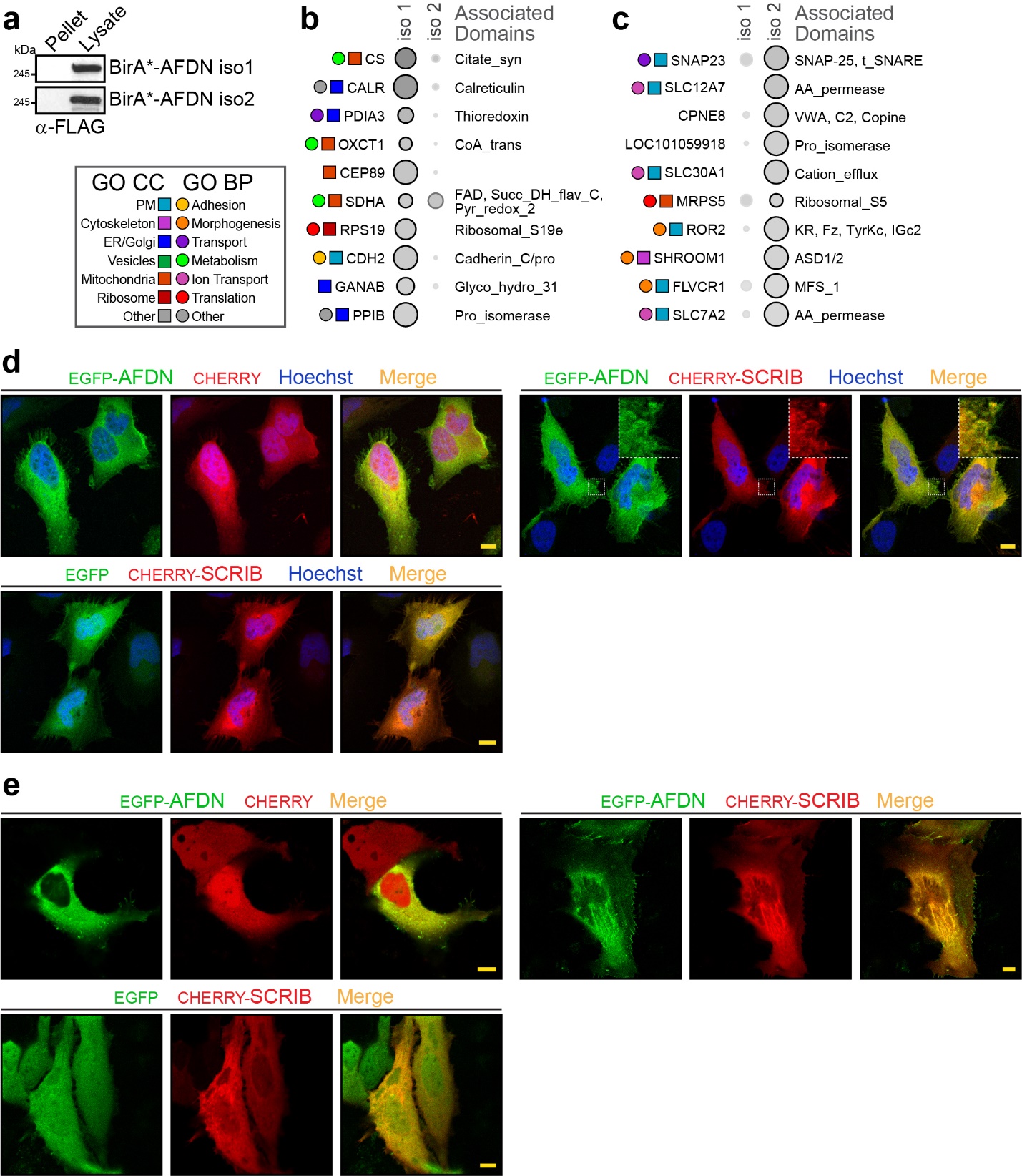


### Supplementary Fig. 1. Isoform-specific preys of AFDN identified by BioID and co-localization of AFDN and SCRIB in HeLa cells. a Tet-inducible BirA*/FLAG-tagged AFDN from stable HeLa cell lines. Tet was added to cells for 24 hours, along with biotin, and Western blotting with anti-FLAG shows expression of the two isoforms of AFDN in the soluble fraction. Source data are provided at the end of Supplementary Information. b The top 10 BioID preys and their associated domains for l-AFDN (iso1) that were not found with s-AFDN (iso2). Available GO CC (square) or GO BP (circle) terms are colored as in the legend (left). The relative abundance, FDR and spectral count are represented by shaded circles and denoted as per Fig. 1d. c The top 10 BioID preys and their associated domains for s-AFDN (iso2) that were not found with l-AFDN (iso1). d AFDN and SCRIB are diffusely distributed in fixed and permeabilized HeLa cells, which lack defined cell-cell contacts and apical-basal polarity. EGFP-AFDN and Cherry-SCRIB are distributed throughout the cytoplasm, with minor enrichment around the cell cortex (*Right*). Cherry or EGFP alone controls are at *Left*. Scale bars represent 10 µm. e AFDN and SCRIB are also diffusely distributed in live HeLa cells. EGFP-AFDN and Cherry-SCRIB are co-distributed around the perinuclear region with minor enrichment around the cell cortex (*Right*). Cherry or EGFP alone controls are at *Left*. Scale bars represent 10 µm.


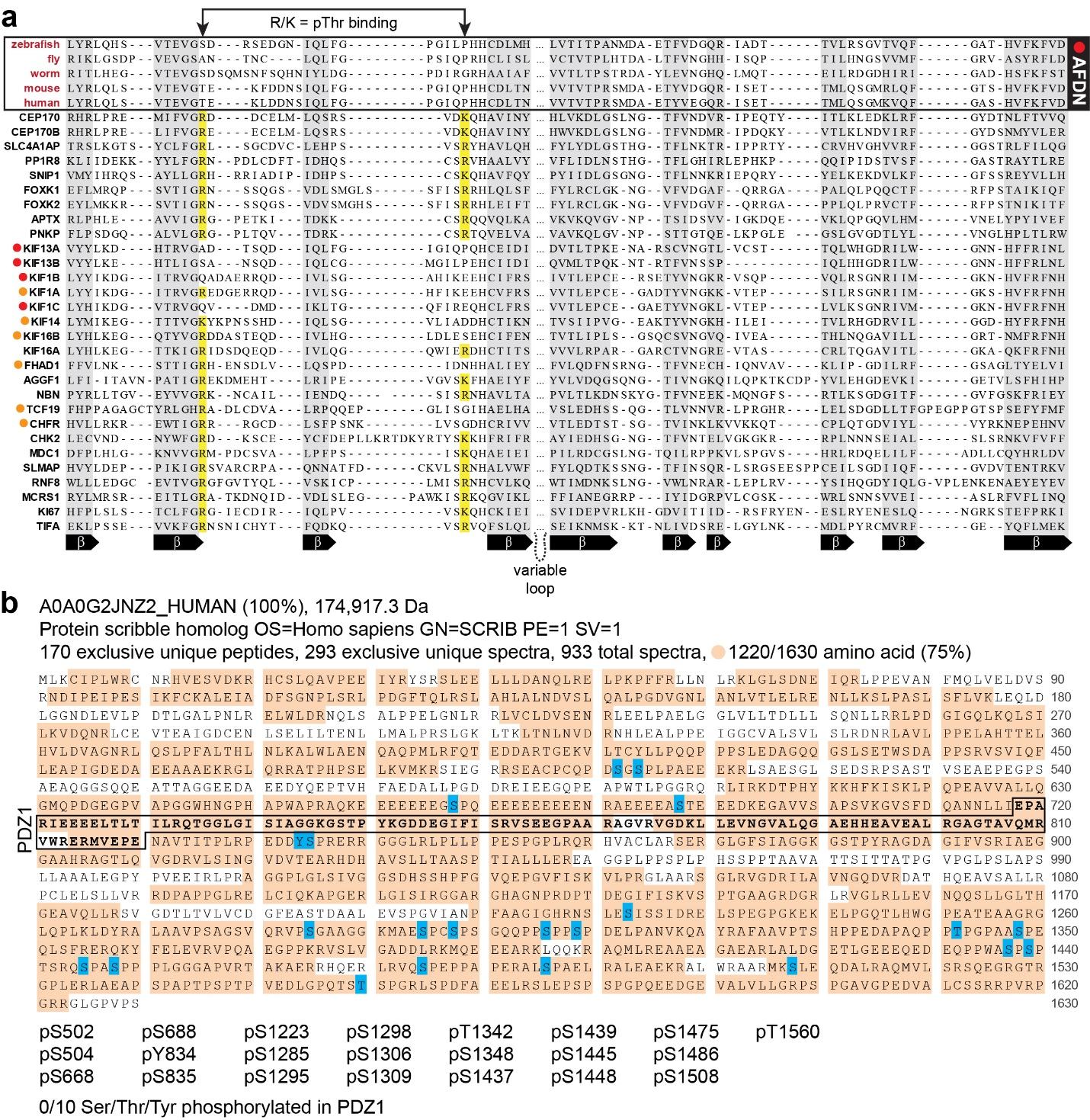


### Supplementary Fig. 2. Sequence alignment of the AFDN FHA domain and phosphoproteomic analysis of SCRIB PDZ1. a Amino acid sequence alignment of all 30 FHA domains in the human proteome, as well as zebrafish, fly, worm and mouse orthologs of AFDN (top, boxed). ARG and LYS residues typically involved in FHA domain interactions with phosphorylated motifs are highlighted yellow. FHA domains missing both positively charged residues are indicated by a red circle, and those missing one by an orange circle. b Phosphorylated residues in SCRIB identified by phosphoproteomics. None are within PDZ1 (boxed). 75% coverage of the protein was attained, including all potential phosphosites in PDZ1.


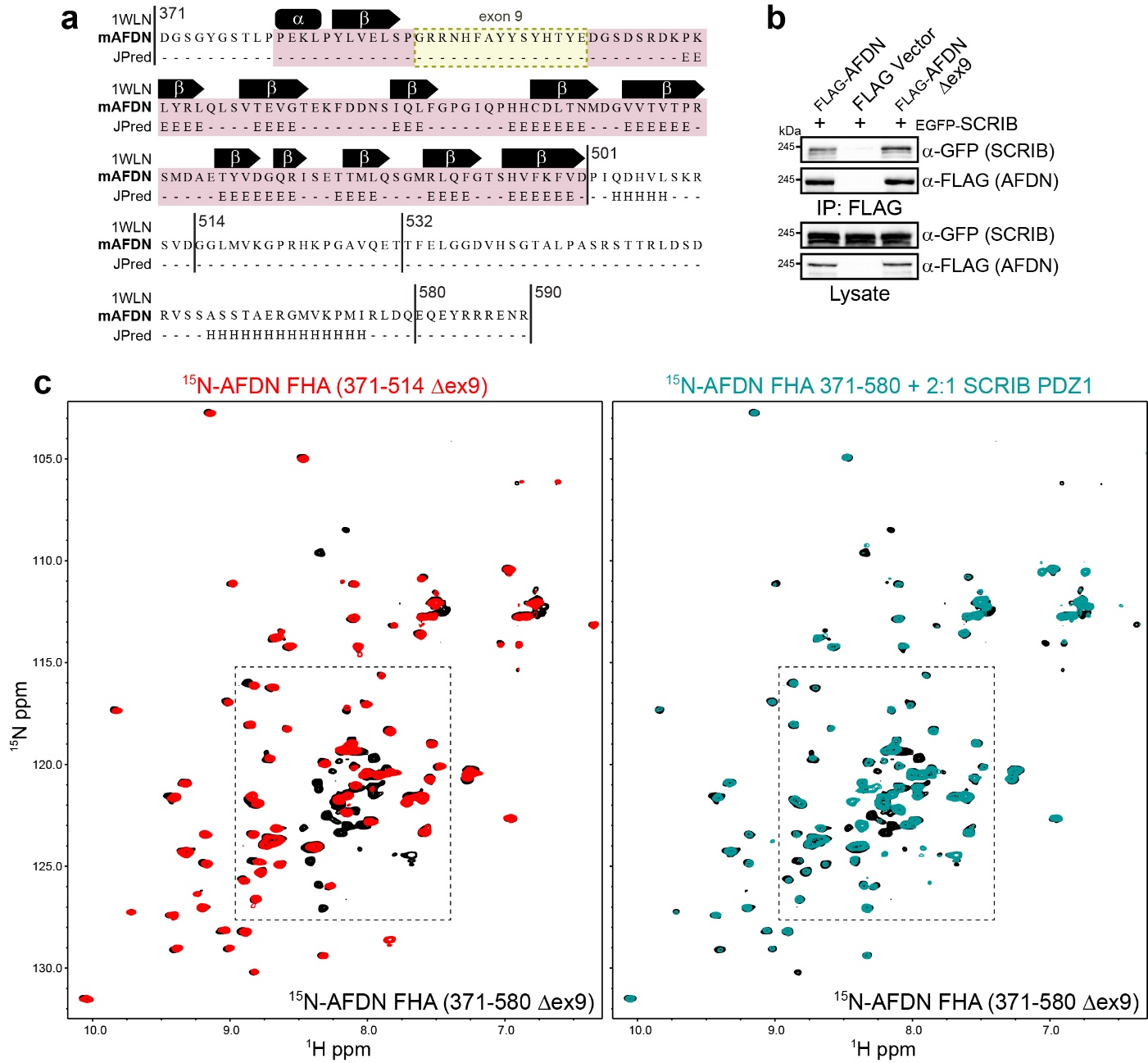


### Supplementary Fig. 3. Analyses of the AFDN FHA-SCRIB PDZ1 interaction. a AFDN is alternatively spliced to generate the l-AFDN and s-AFDN isoforms. A second alternative splice site encompasses exon 9, encoding a fragment of the FHA domain and removes 15 residues from a loop region. Here, secondary structure elements are depicted above the primary sequence of the AFDN FHA domain (derived from structure PDBid 1WLN). Jpred secondary structure predictions are depicted below the amino acid sequence, revealing an additional helical segment from residues 563-576. b Deletion of residues encoding the alternatively spliced exon 9 does not alter the interaction between AFDN and SCRIB. Expression vectors encoding FLAG-tagged AFDN with or without exon 9 residues were co-transfected into HEK 293T cells with EGFP-tagged SCRIB. Following anti-FLAG immunoprecipitation, Western blot with anti-GFP demonstrated no effect of the FHA domain Δexon 9 deletion. Source data are provided at the end of Supplementary Information. c Full ^1^H/^15^N-HSQC spectra of the AFDN FHA domain. *Left*, the extended FHA domain (371-580, black) overlays well with the core FHA domain (371-514, red) and exhibits several additional peaks. The region depicted in Fig. 3e is outlined by a dashed box. *Right*, the same peaks are broadened upon addition of unlabeled SCRIB PDZ1 (blue), verifying importance of the extended C-terminal region to SCRIB binding.


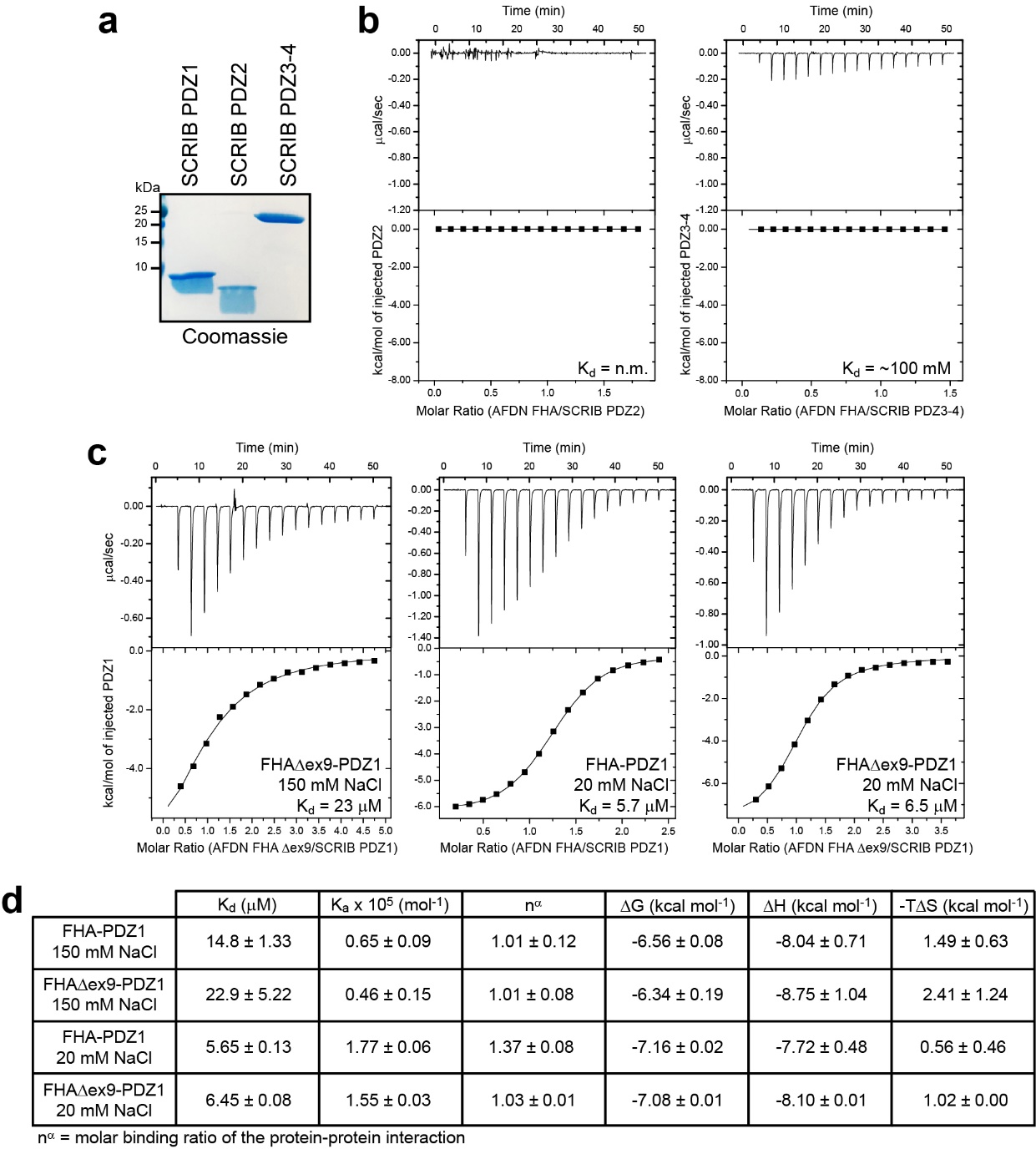


### Supplementary Fig. 4. ITC analysis of AFDN FHA-SCRIB PDZ domain interactions. a SCRIB PDZ domains 1, 2 and the 3-4 supramodule expressed in *E. coli* and purified for ITC. Source data are provided at the end of Supplementary Information. b ITC binding profiles of purified PDZ2 or PDZ3-4 with the AFDN FHA domain. Very weak binding (>100 mM) was observed between the FHA domain and SCRIB PDZ34 (*Right*) and no binding to PDZ2 (*Left*). c ITC binding profiles of SCRIB PDZ1 and the AFDN FHA domain. *Left*, ITC analysis of the FHA-PDZ1 interaction with exon 9 residues deleted from the FHA domain. Experiment was performed with 150 mM NaCl and the derived K_d_ was 23 µM. *Middle*/*Right*, ITC analysis of the FHA-PDZ1 interaction at a lower salt concentration (20 mM NaCl). SCRIB PDZ1 was titrated into the AFDN FHA domain with or without exon 9 residues 393-407 resulting in similar affinities (K_d_ of 5.7 µM for the full FHA domain and 6.5 µM for the Δex9 deletion). d Summary of thermodynamic parameters relating to SCRIB PDZ1/AFDN FHA binding as determined by ITC. Reported is average value from 3 individual replicates +/- the standard deviation.


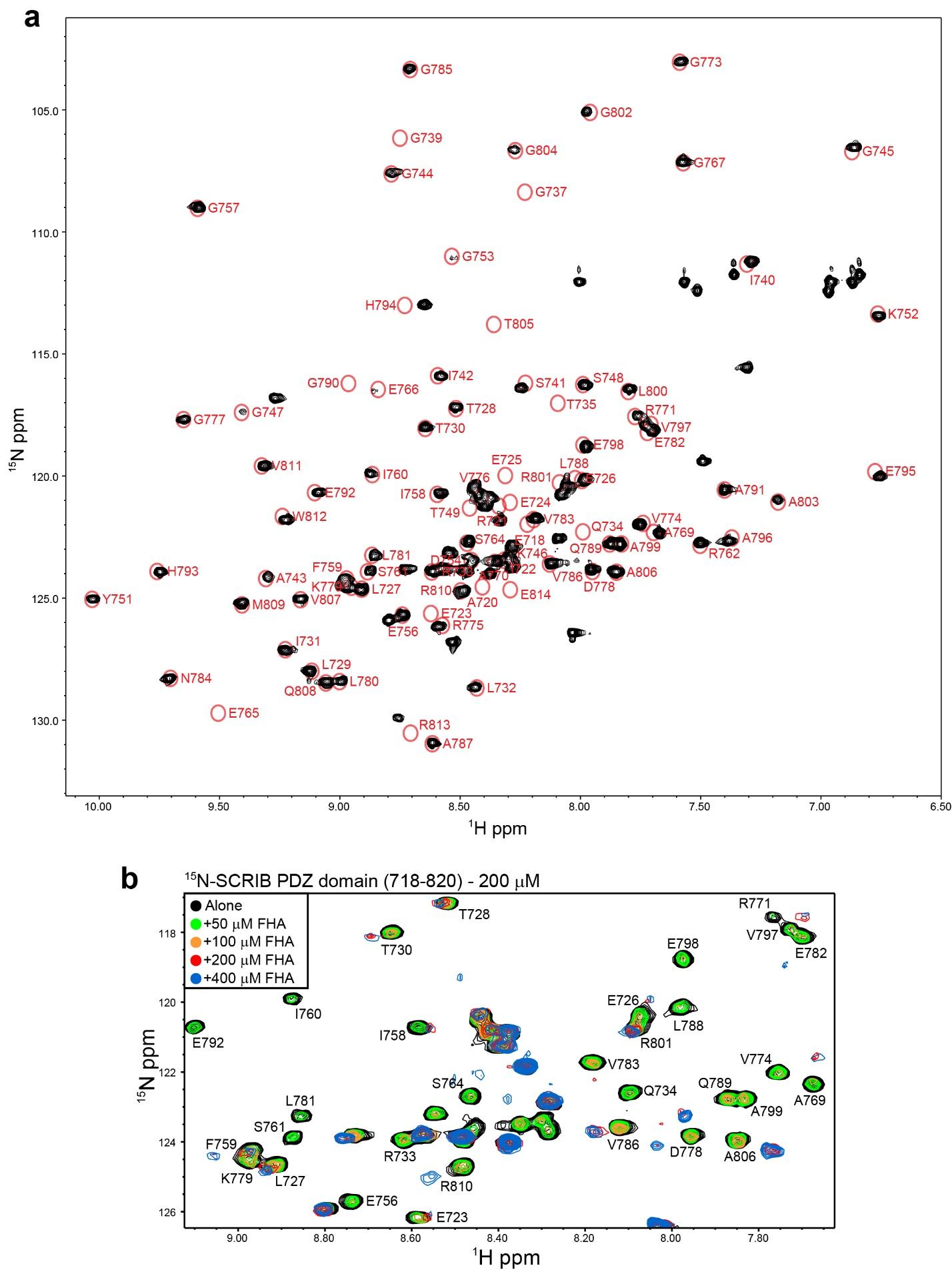


### Supplementary Fig. 5. Biophysical analysis of the AFDN FHA-SCRIB PDZ1 interaction. a Assignment of SCRIB PDZ1 peaks using BMRBid 11207. 71% of the peaks in this ^1^H/^15^N-HSQC of ^15^N-labelled SCRIB PDZ1 could be unambiguously assigned using the available dataset. Peak positions from the BMRB are simulated here, shown in red outline with assigned amino acid displayed adjacent. b Overlay of ^1^H/^15^N-HSQC spectra of the SCRIB PDZ1 domain either unbound (black) or with increasing concentrations of unlabelled AFDN FHA domain (green, orange, red, blue). Exchange broadening or chemical shift perturbations were observed for the majority of peaks. Assigned peaks are labeled by residue.


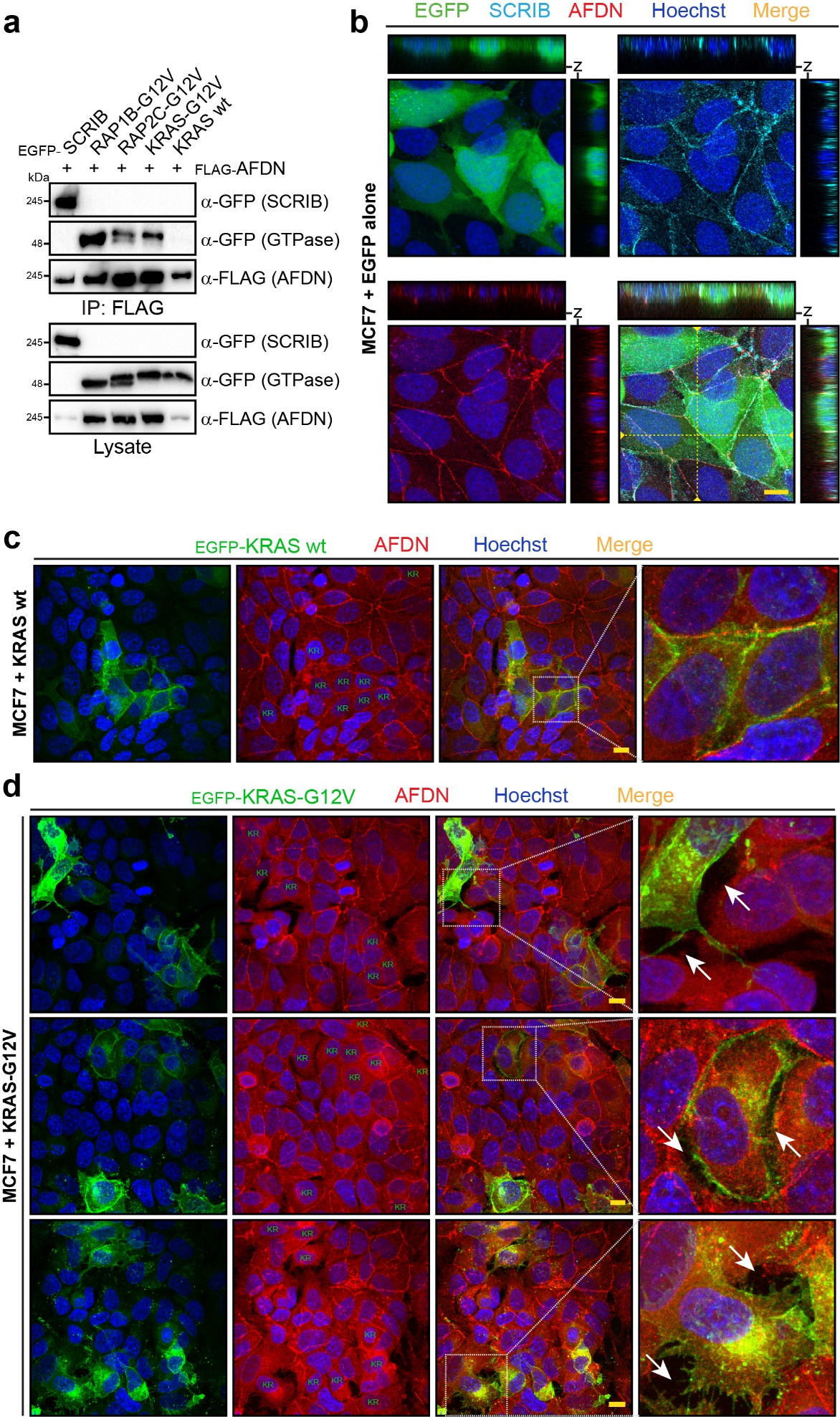


### Supplementary Fig. 6. Interaction of AFDN with RAS small GTPases. a Multiple RAS subfamily GTPases are co-precipitated with full length AFDN. EGFP-tagged, activated GTPase variants or SCRIB were co-expressed with FLAG-AFDN in HEK 293T cells. Anti-FLAG IP followed by an anti-GFP Western blot revealed interacting proteins. Wild-type (GDP-bound) KRAS was a control. Source data are provided at the end of Supplementary Information. b AFDN and SCRIB localization in MCF7 cells expressing EGFP alone. Endogenous AFDN and SCRIB were immunostained and imaged by confocal microscopy. Projections of *z*-stacks are at right and top of the merged images and their position is marked with a dashed yellow line. Scale bars represent 10 µm. c MCF7 cells expressing wild-type KRAS remain predominantly in the monolayer. Following transfection of EGFP-KRAS, cells were starved overnight, fixed and immunostained for endogenous AFDN (red). The intensity of the AFDN signal is amplified to reveal whole cells in the monolayer, and those expressing KRAS wt are marked with ‘KR’. These cells maintain contact with adjacent cells and AFDN staining is preserved between KRAS expressing cells and those not expressing KRAS. Scale bars represent 10 µm. d MCF7 cells expressing KRAS-G12V regularly detach from other cells. Following transfection of EGFP-KRAS-G12V cells were starved, fixed and immunostained for endogenous AFDN (red). The intensity of the AFDN signal is high to reveal monolayer cells and empty space (black). Cells expressing KRAS-G12V are marked with ‘KR’ and typically lose contact with adjacent cells. AFDN staining in these cells is no longer prominent at the membrane. Arrows in fourth column (enlargement of bounded box) highlight empty space surrounding KRAS-G12V expressing cells. Scale bars represent 10 µm


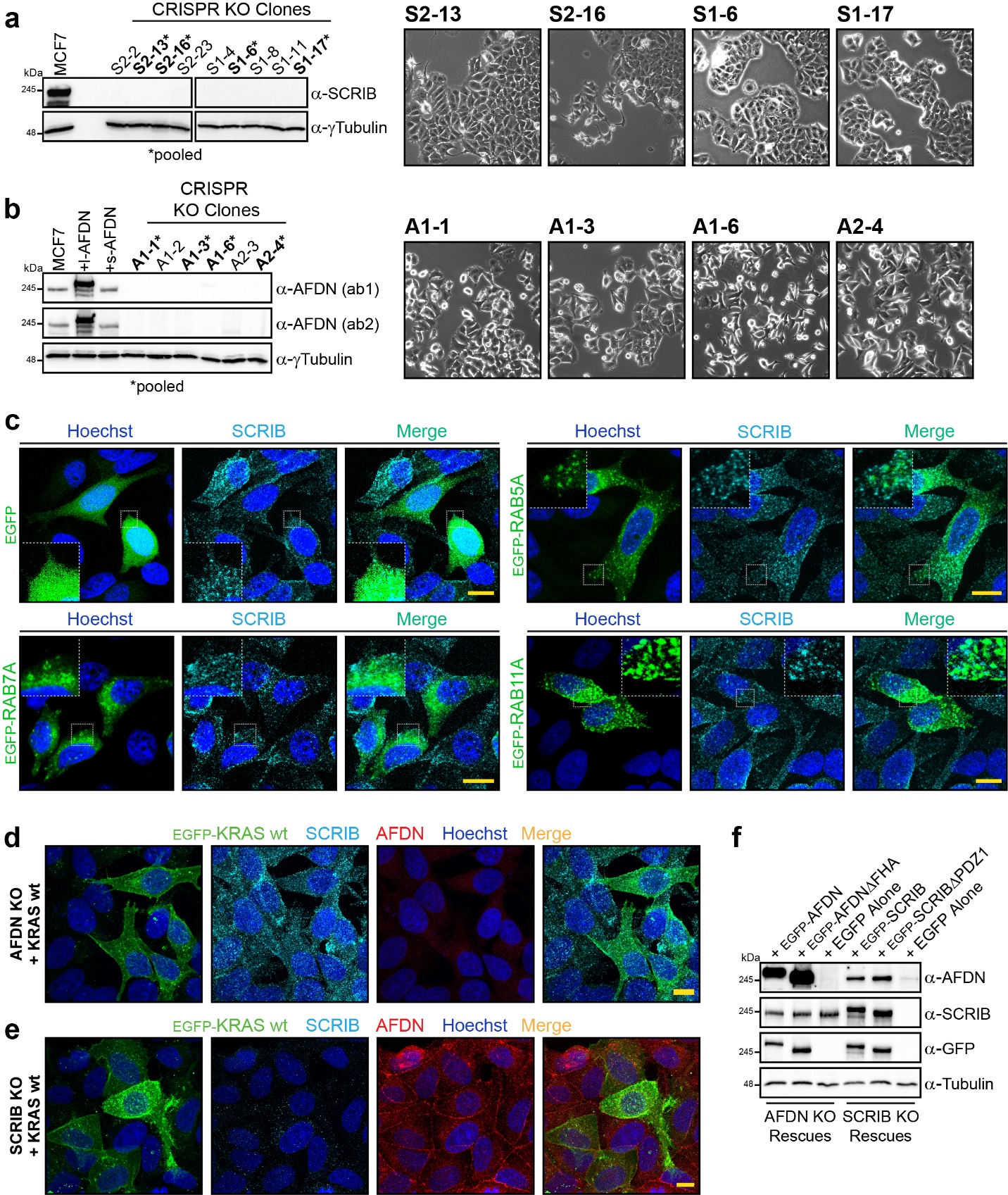


### Supplementary Fig. 7. CRISPR knockout of *AFDN* and *SCRIB* in MCF7 cells. a Western blot of 9 individual *SCRIB* KO clones verified the protein is no longer expressed. “-1” were generated with sgRNA #1 and “-2” with sgRNA #2. Anti-γ-Tubulin was a loading control. The *SCRIB* KO MCF7 clones all exhibit similar phenotypes (right, phase images of 4 clones named at top). The four clones marked with * were mixed to produce the *SCRIB* KO pool. b Western blot of 6 individual *AFDN* KO clones verify the protein is no longer expressed. Two different commercial antibodies were used. “-1” were generated with sgRNA #1 and “-2” with sgRNA #2. Anti-γ-Tubulin was a loading control. The *AFDN* KO MCF7 clones exhibit similar phenotypes (right, phase contrast images of 4 clones named at top). The four clones marked with * were mixed to produce the *AFDN* KO pool. c SCRIB does not localize to endosomes in *AFDN* KO MCF7 cells. EGFP-tagged RAB5A (early), RAB7A (late) or RAB11A (recycling) were used to mark these endosomal pools in *AFDN* KO cells. Staining with anti-SCRIB revealed the characteristic punctate pattern, but the puncta do not overlay with the RAB endosomal markers. Scale bars represent 10 µm. d SCRIB is internalized and demonstrates a punctate pattern in *AFDN* KO MCF7 cells, which do not form cell-cell contacts. Expression of wild-type EGFP-KRAS in these cells does not alter SCRIB localization. Scale bar represents 10 µm. e AFDN remains predominantly at sites of cell-cell contact in *SCRIB* KO MCF7 cells, which grow in multi-cell layers with poorly defined apical-basal polarity. EGFP-KRAS wild-type expression does not alter AFDN localization. Scale bar represents 10 µm. f Rescue of the *AFDN* and *SCRIB* KO lines with lenti-based expression vectors. Cells were transduced with vectors expressing EGFP alone, or EGFP-tagged AFDN, AFDNΔFHA, SCRIB, or SCRIBΔPDZ1 as indicated. Anti-GFP Western blots corroborated expression following selection in G418. All source data are provided at the end of Supplementary Information.


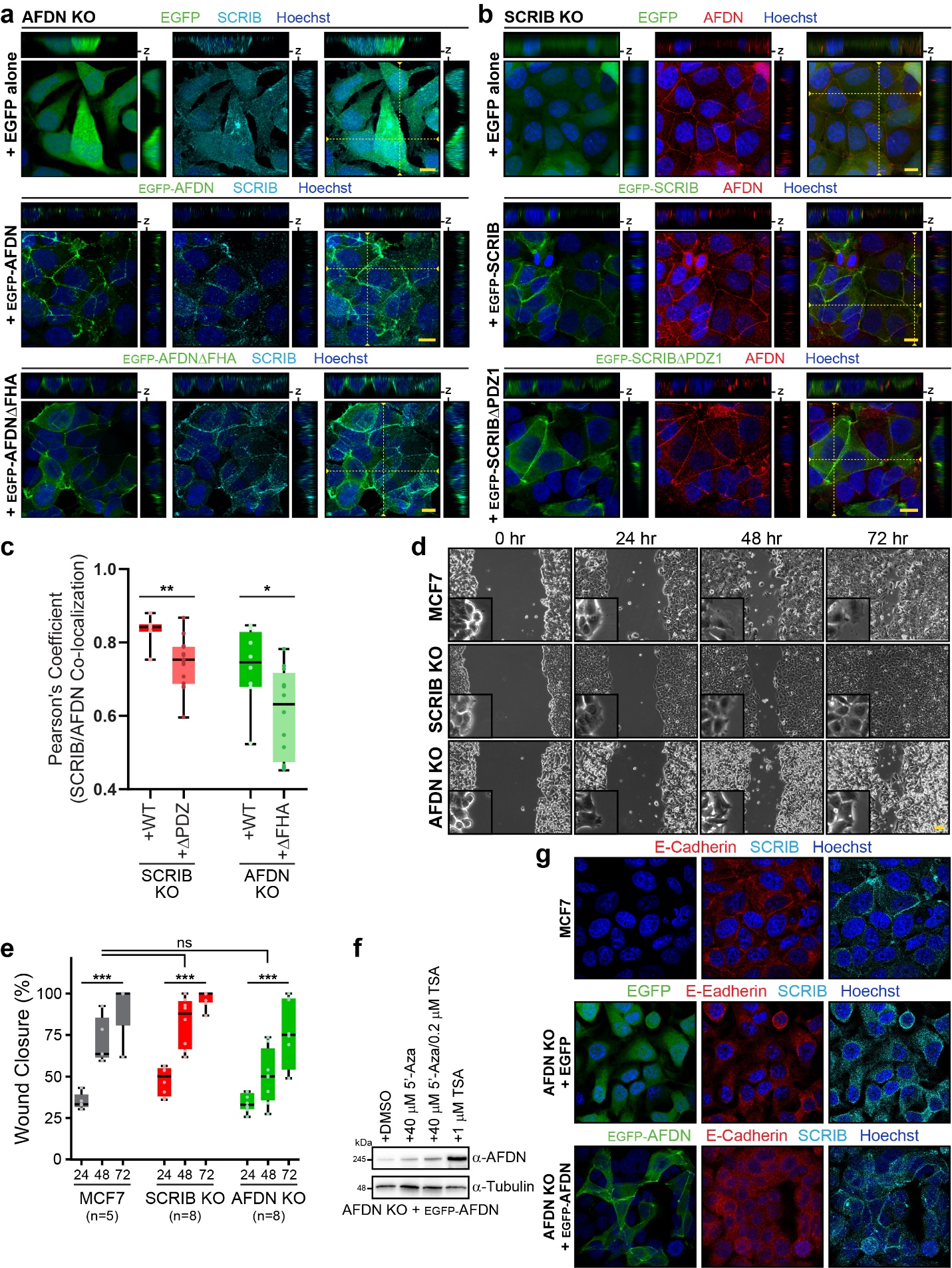


### Supplementary Fig. 8. Rescue and characterization of *AFDN* and *SCRIB* KO cells. a Rescue of *AFDN* KO MCF7 cells by lenti-based expression of EGFP-AFDN. Stable lines expressing EGFP alone or EGFP-AFDNΔFHA were also generated. Confocal images reveal the localization of expressed EGFP proteins, as well as endogenous SCRIB. *z*-stacks are at right and top, at positions indicated by yellow lines in the merged image. Scale bars represent 10 µm. b Rescue of *SCRIB* KO MCF7 cells by lenti-based expression of EGFP-SCRIB. Stable lines were also generated to express EGFP alone or EGFP-SCRIBΔPDZ1. Confocal images show the localization of expressed EGFP proteins, as well as endogenous AFDN. *z*-stacks are at right and top, at positions indicated by yellow lines in the merged image. Scale bars represent 10 µm. c Quantitation of SCRIB/AFDN co-localization in rescued cell lines as determined by Pearson’s coefficient. Multiple *z*-stack projections along regions of cell-cell contact in *AFDN* or *SCRIB* KO MCF7 cells (approximate length of 10 µm each) were used to determine the relative co-localization of endogenous SCRIB with cells expressing wild-type AFDN or the ΔFHA mutant (*AFDN* KO cells; *P*=0.028), or endogenous AFDN with either wild-type SCRIB or the ΔPDZ1 mutant (*SCRIB* KO cells; *P*=0.004). Pearson’s coefficient was calculated using the JACoP plugin for ImageJ. Line represents the median, box the IQR and whiskers min/max as derived n≥7 independent images. ***P*<0.005, **P*<0.05 as measured by unpaired, two-tailed t-test. Source data are provided in the Source Data file. d Phase contrast images of MCF7, *SCRIB* KO and *AFDN* KO cells during wound closure in complete media (10% serum). Inset are enlarged images of cells at the wound edge. Scale bar represents 100 µm. e KO of *SCRIB* or *AFDN* does not significantly alter motility in the presence of serum. Wound closure (%) was measured at 24, 48, or 72 hours for MCF7 (*P*=0.0001 within group), *AFDN* KO (*P*=0.0004) and *SCRIB* KO (*P*=0.0001) cells. Line represents the median, box the IQR and whiskers min/max as derived from n≥5 independent replicates. ****P*<0.001 as measured by RM one-way ANOVA. ns = not significant as determined by two-way ANOVA (*P*=0.1262 for *AFDN* KO and *P*=0.4556 for *SCRIB* KO). Source data are provided in the Source Data file. f AFDN expression is recovered by addition of the histone deacetylase inhibitor TSA. Western blot of AFDN from cells cultured in the presence of TSA, 5`-Aza or DMSO control shows AFDN expression is significantly increased in 1 µM TSA. anti-Tubulin is a loading control. Source data are provided at the end of Supplementary Information. g KO of *AFDN* in MCF7 cells disrupts E-cadherin localization. E-cadherin marks sites of cell-cell contact in the parental MCF7 line (*top*). Endogenous SCRIB is localized to the same regions. In the *AFDN* KO lines, expression of recombinant EGFP-AFDN does not restore E-cadherin at cell-cell contacts, and it remains predominantly in the cytosol (*bottom*). Expression of EGFP alone was a control (*middle*).
