# Supplementary material for: Afadin couples RAS GTPases to the polarity rheostat Scribble
Source: Nat Commun. 2022 Aug 5;13:4562. doi: 10.1038/s41467-022-32335-8 (PMC9355967; doi:10.1038/s41467-022-32335-8)
Supplement: Supplementary file 3 — Description of Additional Supplementary Files [file 41467_2022_32335_MOESM3_ESM.pdf]

Description of Additional Supplementary Information Files Document

Supplementary Dataset 1 - BioID proteomics-identified proteins with in vivo proximity to the short and long isoforms of AFDN.

Supplementary Dataset 2 - Primers used in this study.
