# Supplementary material for: Afadin couples RAS GTPases to the polarity rheostat Scribble
Source: Nat Commun. 2022 Aug 5;13:4562. doi: 10.1038/s41467-022-32335-8 (PMC9355967; doi:10.1038/s41467-022-32335-8)
Supplement: Supplementary file 6 — Reporting Summary [file 41467_2022_32335_MOESM6_ESM.pdf]

Corresponding author(s): SMITH, Matthew J.

Last updated by author(s): Jul 13, 2022

Reporting Summary

Nature Portfolio wishes to improve the reproducibility of the work that we publish. This form provides structure for consistency and transparency in reporting. For further information on Nature Portfolio policies, see our Editorial Policies and the Editorial Policy Checklist.

Statistics

For all statistical analyses, confirm that the following items are present in the figure legend, table legend, main text, or Methods section.

n/a	Confirmed	
☐	☒	The exact sample size (*n*) for each experimental group/condition, given as a discrete number and unit of measurement
☐	☒	A statement on whether measurements were taken from distinct samples or whether the same sample was measured repeatedly
☐	☒	The statistical test(s) used AND whether they are one- or two-sided<br>*Only common tests should be described solely by name; describe more complex techniques in the Methods section.*
☒	☐	A description of all covariates tested
☒	☐	A description of any assumptions or corrections, such as tests of normality and adjustment for multiple comparisons
☐	☒	A full description of the statistical parameters including central tendency (e.g. means) or other basic estimates (e.g. regression coefficient) AND variation (e.g. standard deviation) or associated estimates of uncertainty (e.g. confidence intervals)
☐	☒	For null hypothesis testing, the test statistic (e.g. F, t, r) with confidence intervals, effect sizes, degrees of freedom and P value noted<br>*Give P values as exact values whenever suitable.*
☒	☐	For Bayesian analysis, information on the choice of priors and Markov chain Monte Carlo settings
☒	☐	For hierarchical and complex designs, identification of the appropriate level for tests and full reporting of outcomes
☐	☒	Estimates of effect sizes (e.g. Cohen's *d*, Pearson's *r*), indicating how they were calculated

Our web collection on statistics for biologists contains articles on many of the points above.

Software and code

Policy information about availability of computer code

Data collection	No software used for data collection.
Data analysis	No code was generated. Analysis software included Office (365), Prism (9.3.1), Origin (7.0), ProHits (3.0), SAINTexpress (3.3), Scaffold (5.1.1), ProteoWizard (v3.0.4468), Mascot (v2.3.02), Comet (v2012.02 rev.0), ZEN Blue (2.5), ImageJ (1.53q), ImageLab (5.2.1), NMR Pipe, NMRView (9.2.0-b27) and Bruker TopSpin (4.1.4). All are available for free or commercially.

For manuscripts utilizing custom algorithms or software that are central to the research but not yet described in published literature, software must be made available to editors and reviewers. We strongly encourage code deposition in a community repository (e.g. GitHub). See the Nature Portfolio guidelines for submitting code & software for further information.

Data

Policy information about availability of data

All manuscripts must include a data availability statement. This statement should provide the following information, where applicable:

- Accession codes, unique identifiers, or web links for publicly available datasets
- A description of any restrictions on data availability
- For clinical datasets or third party data, please ensure that the statement adheres to our policy

The mass spectrometry data has been deposited as a complete submission in the ProteomeXchange through partner MassIVE (massive.ucsd.edu), and assigned

identifiers PXD007631 and MSV000081499, respectively. Structures 3UNN [http://doi.org/10.2210/pdb3UNN/pdb], 1WLN [http://doi.org/10.2210/pdb1WLN/pdb], 5VWK [http://doi.org/10.2210/pdb5VWK/pdb] and 1X5Q [http://doi.org/10.2210/pdb1X5Q/pdb] are publicly available in the Protein Data Bank.

Human research participants

Policy information about studies involving human research participants and Sex and Gender in Research.

Reporting on sex and gender	n/a
Population characteristics	n/a
Recruitment	n/a
Ethics oversight	n/a

Note that full information on the approval of the study protocol must also be provided in the manuscript.

Field-specific reporting

Please select the one below that is the best fit for your research. If you are not sure, read the appropriate sections before making your selection.

☒ Life sciences ☐ Behavioural & social sciences ☐ Ecological, evolutionary & environmental sciences

For a reference copy of the document with all sections, see nature.com/documents/nr-reporting-summary-flat.pdf

Life sciences study design

All studies must disclose on these points even when the disclosure is negative.

Sample size	No pre-calculation was performed, sample sizes were increased until the appropriates statistical analyses could be performed.
Data exclusions	No data was excluded.
Replication	All attempts at replication were successful. Every experiment was repeated at least 3 times in independent experiments.
Randomization	n/a - there are no experimental groups in this study for which samples needed to be allocated.
Blinding	Blinding was relevant only with the AFDN/SCRIB rescues and phenotypic experiments. These were performed by trainees and the data analyzed by PI (blinded). No other experiments in this study required an analysis for which bias may alter the results.

Reporting for specific materials, systems and methods

We require information from authors about some types of materials, experimental systems and methods used in many studies. Here, indicate whether each material, system or method listed is relevant to your study. If you are not sure if a list item applies to your research, read the appropriate section before selecting a response.

Materials & experimental systems

n/a	Involved in the study	
☐	☒	Antibodies
☐	☒	Eukaryotic cell lines
☒	☐	Palaeontology and archaeology
☒	☐	Animals and other organisms
☒	☐	Clinical data
☒	☐	Dual use research of concern

Methods

n/a	Involved in the study	
☒	☐	ChIP-seq
☒	☐	Flow cytometry
☒	☐	MRI-based neuroimaging

Antibodies

Antibodies used	Antibodies used were: anti-AFDN polyclonal (Abcam, ab203569; WB: 1:500), anti-AFDN monoclonal (Abcam, ab90809/clone 3; WB: 1:200, IF: 1:100), anti-SCRIB (Cell Signalling, 4475S; WB: 1:1000, IF: 1:50), anti-γ-Tubulin (Sigma, T6557/clone GTU-88; WB: 1:5000), anti-Myc (Roche, 11667149001), anti-GFP (Abcam, ab290; WB: 1:5000), anti-FLAG M2 (Sigma, F3165; WB: 1:1000, IF: 1:200), anti-E-Cadherin (BD, 610181/clone 36; WB: 1:5000), anti-phospo-ERK1/2 (Cell Signalling, 9101; WB: 1:1000), anti-phospho-AKT (Cell Signalling, 4060; WB: 1:1000), anti-ERK1/2 (Cell Signalling, 4695; WB: 1:1000) anti-AKT (Cell signalling, 4691; WB: 1:1000), anti-mouse

	Tx-Red (Sigma, SAB3701076; WB: 1:100), anti-Rabbit Alexa Fluor 647 (Life Technologies, A-21244; WB: 1:200), anti-rabbit Cy5 (Abcam, ab6564; WB: 1:200), anti-rabbit Alexa Fluor 488 (Life Technologies, A-27034; WB: 1:200), HRP-conjugated anti-rabbit (Cedarlane, NA934; WB: 1:10000) and HRP-conjugated anti-mouse IgG (Fisher, 45-000-679; WB: 1:10000).
Validation	Only relevant validation of primary antibodies was for anti-AFDN and anti-SCRIB These were validated by our own CRISPR knockout of the proteins (this study, confirmed by Sanger sequencing) in the human breast cancer epithelial line MCF7. We have further validated these with CRISPR KO of these proteins in the human breast line MCF10A. There are no validation statements on the manufacturers website.

Eukaryotic cell lines

Policy information about cell lines and Sex and Gender in Research

Cell line source(s)	Human embryonic kidney epithelial (HEK 293T; ATCC CRL-3216), human cervix epithelial (HeLa; ATCC CCL-2), human mammary epithelial (MCF7; ATCC HTB-22), canine kidney epithelial (MDCKII; ATCC CRL-2936) were from ATCC. HeLa Flp-In T-REx line was from Thermo (R71407).
Authentication	Not re-authenticated.
Mycoplasma contamination	All cell lines consistently tested negative for mycoplasma contamination.
Commonly misidentified lines (See ICLAC register)	None.
